# Epigallocatechin-3-gallate and 6-OH-11-O-Hydroxyphenanthrene Limit BE(2)-C Neuroblastoma Cell Growth and Neurosphere Formation In Vitro

**DOI:** 10.3390/nu10091141

**Published:** 2018-08-22

**Authors:** Fulvia Farabegoli, Marzia Govoni, Enzo Spisni, Alessio Papi

**Affiliations:** 1 Department of Pharmacy and Biotechnology (FaBiT), University of Bologna, 40126 Bologna, Italy; 2 Department of Experimental, Diagnostic and Specialty Medicine (DIMES), University of Bologna, 40138 Bologna, Italy; marzia.govoni@unibo.it; 3 Department Biological, Geological, and Environmental Sciences (BiGeA), University of Bologna, 40126 Bologna, Italy; enzo.spisni@unibo.it (E.S.); alessio.papi2@unibo.it (A.P.)

**Keywords:** EGCG, 6-OH-11-O-hydroxyphenanthrene, neuroblastoma, BE(2)-C, N-MYC, neuro-sphere

## Abstract

We conducted an in vitro study combining a rexinoid, 6-OH-11-O-hydroxyphenanthrene (IIF), and epigallocatechin-3-gallate (EGCG), which is the main catechin of green tea, on BE(2)-C, a neuroblastoma cell line representative of the high-risk group of patients. Neuroblastoma is the most common malignancy of childhood: high-risk patients, having N-MYC over-expression, undergo aggressive therapy and show high mortality or an increased risk of secondary malignancies. Retinoids are used in neuroblastoma therapy with incomplete success: the association of a second molecule might improve the efficacy. BE(2)-C cells were treated by EGCG and IIF, individually or in combination: cell viability, as evaluated by 3-(4,5-dimethylthiazol-2-yl)-2,5-diphenyl tetrazolium bromide (MTT) assay, was reduced, EGCG+IIF being the most effective treatment. Apoptosis occurred and the EGCG+IIF treatment decreased N-MYC protein expression and molecular markers of invasion (MMP-2, MMP-9 and COX-2). Zymography demonstrated nearly 50% inhibition of MMP activity. When BE(2)-C cells were grown in non-adherent conditions to enrich the tumor-initiating cell population, BE(2)-C-spheres were obtained. After 48 h and 72 h treatment, EGCG+IIF limited BE(2)-C-sphere formation and elicited cell death with a reduction of N-MYC expression. We concluded that the association of EGCG to IIF might be applied without toxic effects to overcome the incomplete success of retinoid treatments in neuroblastoma patients.

## 1. Introduction

Neuroblastoma is the most common malignancy of childhood, arising from embryonic sympathetic neural cell precursors and accounting 12% of cancer deaths in children younger than 15 years of age. Neuroblastoma is a heterogeneous disease: patients belonging to low- or intermediate-risk groups have excellent long-term survival, whereas patients harboring a high-risk phenotype, which are characterized by widespread disease dissemination, show long-term survival rates below 50%. In high-risk patients, complete clinical remission is often followed by relapse and fatal outcome, possibly because of the persistence of neoplastic cells (minimal residual disease). Furthermore, high-risk patients are treated aggressively with chemotherapy, radiation, surgery, and myeloablative and immunotherapies, thus giving rise to short- and long-term toxicity. The current intensive therapeutic strategy is associated with an 18-fold increased risk of secondary neoplastic disease, mainly acute myelogenous leukemia [1,2]. For these reasons, more effective and less toxic treatments are needed in addition to the development of targeted therapies [3]. 

Retinoids are Vitamin A derivatives that include all-*trans*-retinoic acid (ATRA), 13-*cis*-retinoic acid, (13cRA), and fenretinide (4-HPR). Retinoic acid is one of the most effective differentiation inducers of neuroblastoma cells in vitro. Both ATRA and 13cRA can cause the arrest of cell growth and induce morphological differentiation of human neuroblastoma cell lines [4]. Clinical trials also demonstrated significantly improved survival in high-risk neuroblastoma patients. To control minimal residual disease, high-risk neuroblastoma patients are currently treated with the differentiating agent 13cRA at the completion of cytotoxic therapy (myeloablative therapy, followed by autologous hematopoietic stem cell transplantation), leading to a three-year disease-free survival rate in nearly 50% of patients [5,6]. Differences in the pharmacokinetic properties of 13cRA and ATRA (higher peak levels and a much longer half life for 13cRA with respect to ATRA) make 13cRA the best molecule for use in high-risk neuroblastoma [6].

Retinoids are usually well tolerated with minimal side-effects. However, certain high-risk cohorts, such as patients with N-MYC-amplified neuroblastoma, are innately resistant to retinoid therapy [7]. N-MYC is a neuronal-specific member of the MYC proto-oncogene family, which is expressed during normal neural crest development. Under normal regulation, N-MYC does not prevent terminal differentiation of neuroblasts, whereas aberrant N-MYC signaling alone is sufficient to induce neuroblastoma in animal models [8]. N-MYC amplification occurs in over 20% of neuroblastomas: both copy number increase and overexpression are the strongest negative prognostic factors in neuroblastoma [9]. N-MYC amplification contributes to metastasis, chemoresistance, and resistance to retinoic acid (RA) therapy [10]. The reasons for resistance are unknown, but recent results demonstrated that N-MYC and retinoic acid (RA) are antagonist regulators, with N-MYC overexpression preventing the normal transcriptional response to RA [7].

Preclinical and clinical data support the use of single drugs inhibiting multiple molecular targets or combination therapies involving multiple drugs to achieve greater antineoplastic activity and overcome drug resistance [11]: inhibition of multiple signaling pathways is emerging as a new paradigm for anticancer treatment. Our previous studies on human carcinoma cell lines demonstrated that when the synthetic retinoid 6-OH-11-O-hydroxyphenanthrene (IIF) was associated with epigallocatechin-3-gallate (EGCG), the most active catechin being present in green tea, cytotoxicy increased and molecules that were related to invasion were downregulated [12,13,14]. IIF was more effective than ATRA in arresting cell growth and differentiation in neuroblastoma cells [15]. As retinoid treatments were found to be synergistic with flavonoids, including EGCG [16,17], we investigated the cytotoxic effects of the EGCG plus IIF combination and the molecular network underlying the cytotoxic effects in a neuroblastoma cell line BE(2)-C, a clone of the SK-N-BE(2) neuroblastoma cell line having N-MYC amplification and p53 mutation and isolated from a bone marrow biopsy that was taken in a neuroblastoma patient after repeated courses of chemotherapy and radiotherapy. We also investigated the effects of EGCG and IIF on neurosphere formation. The neurosphere is considered a subpopulation of tumor-initiating cells (also called cancer stem cells) showing the capacity for self-renewal, multipotency, and tumor maintenance [18]. These cells are thought to play a central role in tumor initiation and progression, resistance to therapy, and metastasis formation, and to be primarily responsible for relapse and poor outcome.

## 2. Materials and Methods 

### 2.1. Cell Lines

BE(2)-C cell line was purchased from the American Type Culture Collection (Rockville, MD, USA) and grown in Dulbecco’s modified Eagle’s medium (DMEM) (Sigma-Aldrich, St. Louis, MO, USA), supplemented with 10% fetal calf serum (FCS, Euroclone, Milan, Italy), 2 mM l-glutamine, 50 U/mL penicillin, and 50 μg/mL streptomycin in a humidified atmosphere with 5% CO_2_. Cell lines were routinely tested for mycoplasma infection by fluorescence microscope inspection after 4′,6-diamidino-2-phenylindole (DAPI) staining.

### 2.2. Neurosphere Formation Assay

The neurosphere assay is the gold standard method for studying normal and cancer stem cells. BE(2)-C cells (1 × 10^5^) were grown in low attachment 24-well plates in DMEM/F12, supplemented with 40 ng/mL FGF, 20 ng/mL GF, B27 and 500 U/mL of penicillin/streptomycin. EGCG (20 μg/mL, corresponding to 43.6 μM) or IIF (10 μM) or the EGCG + IIF combination were dissolved in the medium to evaluate sphere formation. After 48 h and 72 h, the spheres were mechanically disaggregated and the cells were collected, centrifuged, stained by trypan blue, and then counted. Some cells were also used for RNA isolation and RT-PCR.

### 2.3. Reagents

EGCG, l-glutamine, penicillin-streptomycin, 3-(4,5-dimethylthiazol-2-yl)-2,5-diphenyl tetrazolium bromide (MTT), 4′,6-diamidino-2-phenylindole (DAPI), 1,4-diazabicyclo(2.2.2)ctane (DABCO), basic fibroblast growth factor (bFGF), and epidermal growth factor (EGF) were all purchased by Sigma-Aldrich, St. Louis, MO, USA. B27 was provided by Thermo-Fisher, Waltham, MA, USA. 6-OH-11-O-hydroxyphenanthrene (IIF) (pat. WIPO W0 00/17143) was provided by K. Ammar, Houston, TX USA. E-MEM, Dulbecco’s modified Eagle’s medium (D-MEM), D-MEM/nutrient mixture F-12 (DMEM/F12), and FBS were purchased by Euroclone, Milan, Italy. Formalin (40%) was from Carlo Erba, Milan, Italy. Antibodies: anti-RARα and anti-RXRγ (Tema Ricerca, Bologna, Italy), anti-EGFR (Thermo Scientific, Waltham, MA, USA), anti-p1068EGFR (Novex, Life Technologies, Carlsbad, CA, USA), anti-Bcl-2 (Sigma-Aldrich, St. Louis, MO, USA), anti-Bax (Applied Biosystem, Monza, Italy) anti-PARP (Santa Cruz Biotechnology, Dallas, TX, USA), anti-COX-2 (Sigma-Aldrich, St. Louis, MO, USA), anti-N-MYC, anti MMP-2, MMP-9, and anti-TIMP-1 (all from Santa Cruz Biotechnology, Dallas, TX, USA), anti-β-tubulin (Sigma-Aldrich, St. Louis, MO, USA), anti-rabbit, and anti-mouse peroxidase conjugated antibodies (GE Healthcare, Milan, Italy).

### 2.4. EGCG and IIF Treatments

EGCG (10 mg/mL tock solution, stored at −20 °C) and IIF (780 μM in polyethylene glycol, stored at 4 °C) were dissolved in complete DMEM medium before treatments. EGCG concentrations from 5 to 20 μg/mL (corresponding to 10.9 μM−43.6 μM) and IIF 5−20 μM were used.

### 2.5. MTT Assay

Cells (20,000/well) were plated in triplicate in a 96-well plate and then incubated with EGCG and IIF, alone and in combination at the defined concentrations for 24, 48, and 72 h. The medium was removed and cells were washed with phosphate buffered saline (PBS). MTT (dissolved in PBS) was diluted in fresh complete medium to a final concentration of 0.5 mg/mL and then incubated at 37 °C. After 3 h, the medium was removed and 100 μL dimethyl sulfoxide (DMSO) was added. After 1 h (at room temperature) the purple formazan crystals were dissolved and the plates were read in a microplate reader (Bio-Rad, Hercules, CA, USA). Absorbance was set at 570 nm. The results were expressed as a percentage of treated on control samples (untreated cells).

### 2.6. Combination Index (CI)

Synergistic, additive, or antagonistic effects after EGCG and IIF treatments were evaluated by the combination index (CI) method, as previously reported [13]. Briefly, C > 1 indicates Antagonism, C = 1 indicates Additivity and C < 1 indicates Synergism.

### 2.7. RNA Isolation

RNA was isolated with PureZOL™ RNA Isolation Reagent (Bio-Rad laboratories, Berkeley, CA, USA), according to the manufacturer’s specifications. 

### 2.8. Reverse Transcriptase-Polymerase Chain Reaction (RT-PCR)

RT-PCR was performed by one-step RT-PCR kit from Thermo Fisher Scientific (Waltham, MA, USA), which enables retrotranscription and cDNA amplification to occur in a single step. β-actin was used as a housekeeping gene and the primers were added to the target gene primers in the same tube. The primer sequences are reported in Table S1. PCR products were loaded onto a 2% agarose gel, run in an electrophoresis chamber, stained by ethidium bromide, and visualized with a UV transilluminator. Bands were analyzed by Kodak Electrophoresis Detection and Analysis System (EDAS 290) (Eastman Kodak Company, Rochester, NY, USA).

### 2.9. Quantitative Polymerase Chain Reaction (qPCR)

Real Time Quantitative analysis of cDNA was performed using a fluorescent nucleic acid dye that was similar to SYBR Green (SsoFast^TM^ EvaGreen Supermix, BioRad Laboratories Inc., Hercule, CA, USA) in a CFX96 system (BioRad Laboratories Inc., Hercule, CA, USA). Primer sequences are reported in Table S2. We used the 2^−ΔΔ*C*t^ method for relative quantification of gene expression.

### 2.10. Western Blot

The cells were treated with EGCG and/or IIF for 24 h or 48 h and then dissolved in lysis buffer as previously described [19]. Cell lysates were size-fractioned in 10–12% SDS-polyacrylamide before transfer to Hybond TM-C Extra membranes (GE Healthcare, Buckinghamshire, UK). Membranes were blocked and incubated overnight at 4 °C with the antibodies. Anti-EGFR, anti-p 1068EGFR, anti-Bax, anti-Bcl2, anti-PARP, anti-RARα and anti-RXRγ, anti-MMP2, anti-MMP9, anti-COX-2, and anti-TIMP1 were diluted 1:500 and the anti-rabbit/mouse peroxidase conjugated antibodies (GE Healthcare, Buckinghamshire, UK) were diluted 1:1000. Bands were quantified by using densitometric image analysis software (Image Master VDS, Pharmacia Biotech, Sweden). Protein loading was controlled by anti-actin or anti-tubulin (1:1000) (both from Sigma-Aldrich, St. Louis, MO, USA) detection. Experiments were performed in triplicate, normalized against actin or tubulin control, and statistically evaluated. Stripping solution (Pierce, Waltham, MA, USA) was used to reprobe the same membranes.

### 2.11. Zymography

Cells were seeded and after 18 h were placed in serum-free medium (D-MEM) with EGCG or IIF or both for 24 h. MMP2 and MMP9 activity was determined by gelatine zymography, as previously described [12]. The MMP activities, indicated by clear bands of gelatin digestion on a blue background, were quantified by using densitometric image analysis software (Image Lab Master, Hercules, CA, USA).

### 2.12 Statistical Analysis

Statistical significance was assessed by ANOVA multiple comparison test with standard deviation (SD), as appropriate, while using PRISM 5.1 (GraphPad, La Jolla, CA, USA). The level for accepted statistical significance was *p* < 0.05. 

## 3. Results

### 3.1. RAR and RXR Expression Changed after EGCG and IIF Treatments 

**Figure 1:**
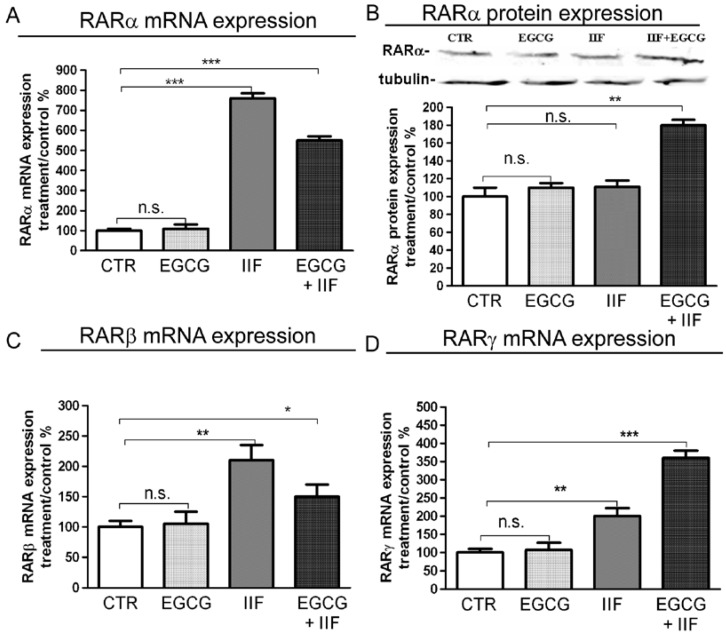
RARα β and γ mRNA expression and RARα protein expression in BE(2)-C neuroblastoma cells. Cells were treated with 20 μg/mL epigallocatechin-3-gallate (EGCG) and 10 μM 6-OH-11-O-hydroxyphenanthrene (IIF), individually and in combination for 24 h. (**A**,**C**,**D**) RARα β and γ mRNA expression as detected by qPCR in control (CTR) and treated samples. (**B**) RARα protein expression. Proteins (50 μg) from total cell lysates were subjected to Sodium Dodecyl Sulphate-PolyAcrylamide Gel Electrophoresis SDS–PAGE and Western blot analysis. The values were normalized to the untreated controls. β-tubulin was used as a loading control. The results are expressed as the average ± standard errors (SE) of three independent experiments. * *p* < 0.05; ** *p* < 0.01; *** *p* < 0.001. n.s.: not significant.

**Figure 2:**
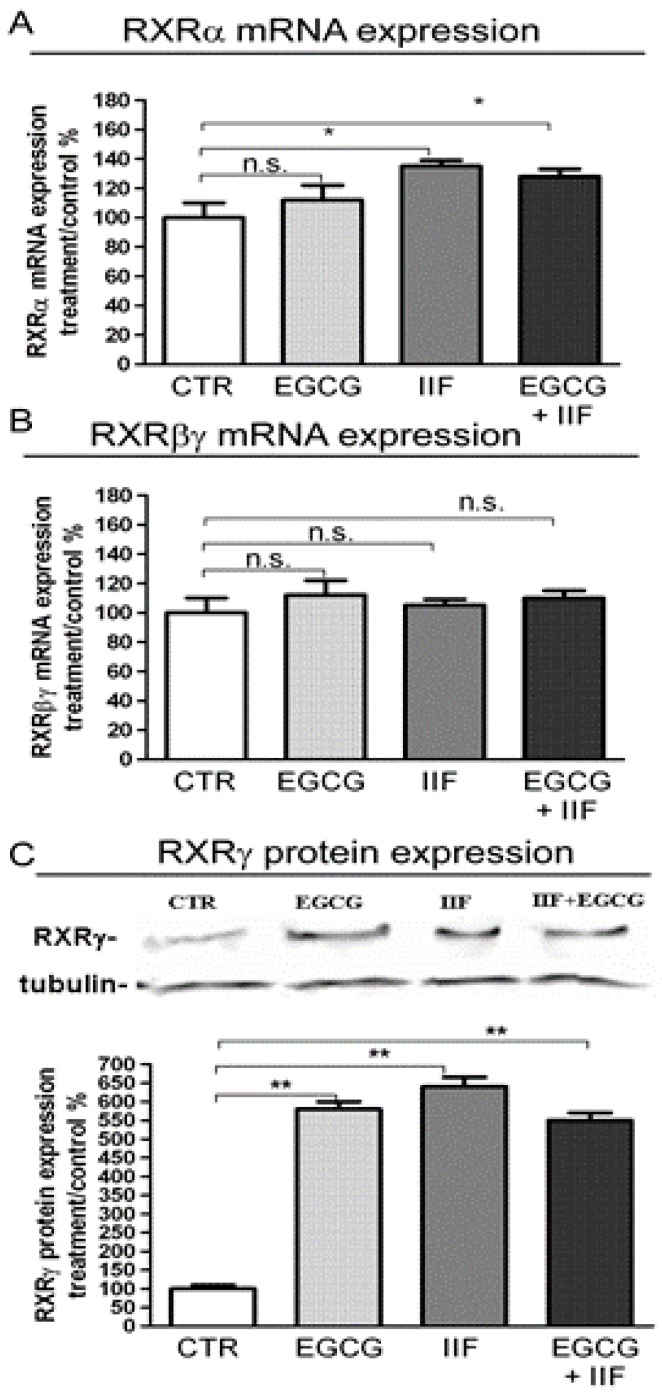
RARα β and γ mRNA expression and RXRγ protein expression in BE(2)-C neuroblastoma cells. Cells were treated with 20 μg/mL l EGCG and 10 μM 6-OH-11-O-hydroxyphenanthrene (IIF), individually and in combination for 24 h. RXRα (**A**) and RXRβγ (**B**) mRNA expression as detected by qPCR in control (CTR) and treated samples. (**C**) RXRγ protein expression. Proteins (50 μg) from total cell lysates were subjected to SDS–PAGE and Western blot analysis. The values were normalized to the untreated controls. β-tubulin was used as a loading control. The results are expressed as the average ± SE of three independent experiments. * *p* < 0.05; ** *p* < 0.01. n.s.: not significant.

Initiation of the retinoid signal is believed to require the formation of RAR-RXR protein heterodimers in the promoter regions of the retinoid target genes. We examined RAR and RXR mRNA expression and protein changes after individual and combined treatments. EGCG alone did not elicite any change in RARα, β or γ in BE(2)-C cells, whereas IIF, alone and/or in combination with EGCG, raised RARα, β or γ mRNA expression a hundredfold. As RARα was the main RAR isoform expressed in BE(2)-C cells (about 80%, data not shown) and mRNA expression was so greatly increased, we only investigated RARα protein expression by Western blot. We found that RARα protein expression nearly doubled in EGCG+IIF-treated samples (Figure 1B). RXR and particularly RXRγ are the main targets of IIF: as we found that RXRγ mRNA was hardly detectable (data not shown), we used a primer couple that is able to cover a homology region in RXRβ and γ genes, a strategy that we already applied elsewhere [12]. As expected, RXRα and βγ RNA expression augmented after IIF treatment and RXRγ protein expression increased significantly after all treatments (Figure 2). Interestingly, EGCG individual treatment significantly enhanced RXRγ protein expression (Figure 2C). PPARs are potential RXR ligands that are able to elicit a response in neuronal cells, including neuroblastoma cells: EGCG+IIF treatment increased PPARβ expression in BE(2)-C cells (Figure S1). In addition, the retinoid-dependent signaling was triggered by IIF when it was given alone and/or in combination with EGCG.

### 3.2. Synergistic Effect of EGCG and IIF in Combination Enhanced Cytotoxicity and Activated Apoptosis in BE(2)-C Cells

**Figure 3:**
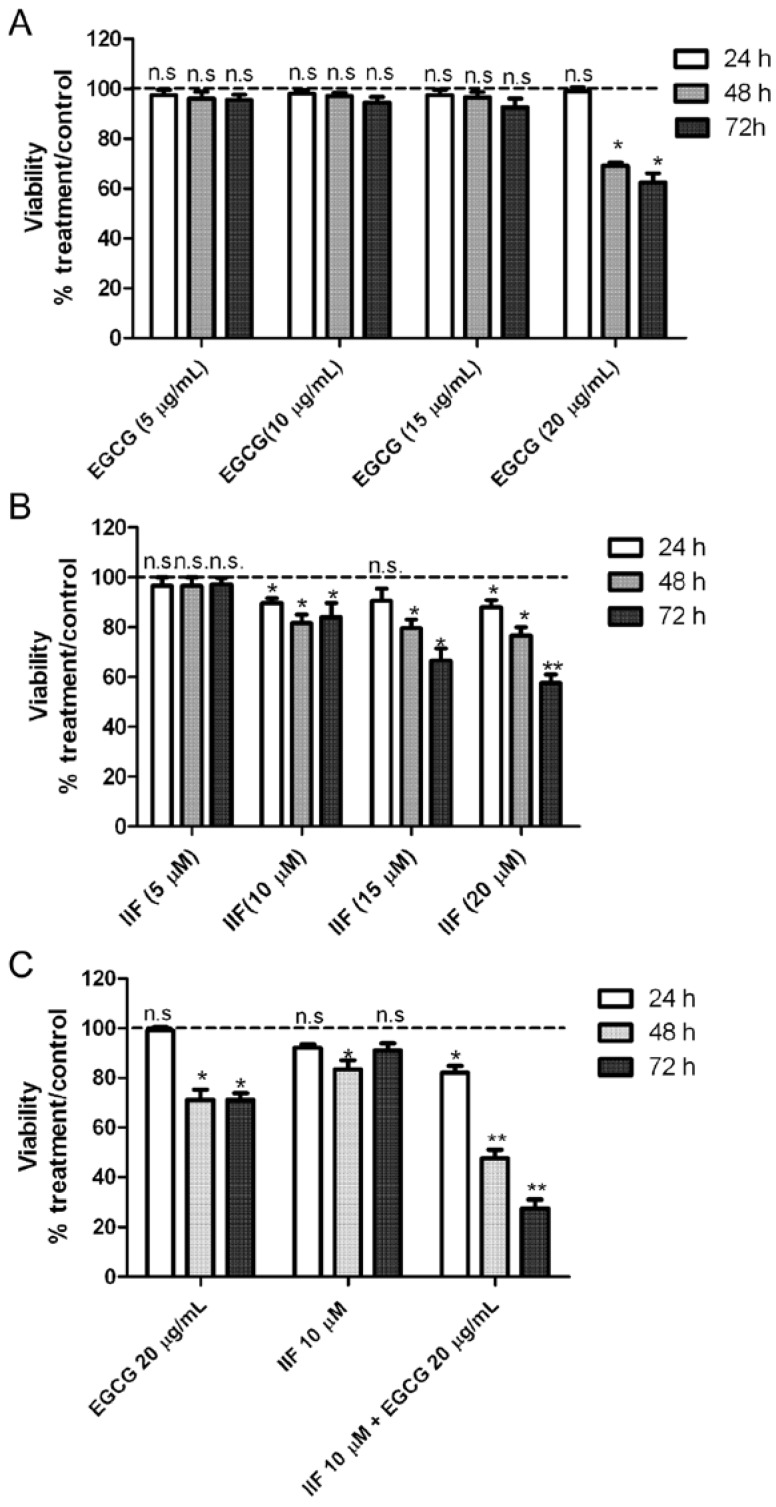
Inhibitory effects of EGCG and IIF treatments on BE(2)-C neuroblastoma cell growth as evaluated by 3-(4,5-dimethylthiazol-2-yl)-2,5-diphenyl tetrazolium bromide (MTT) assay. (**A**) EGCG cytotoxicity time-course (24 h–48 h–72 h) at different concentrations (5, 10, 15, 20 μg/mL). (**B**) IIF cytotoxicity time-course (24 h–48 h–72 h) at different concentrations (5, 10, 15, 20 μM). (**C**) EGCG (20 μg/mL) and IIF (10 μM) (EGCG+IIF) combination. The results are expressed as the average ± SE of three independent experiments. * *p <* 0.05; ** *p <* 0.01. n.s.: not significant.

**Figure 4:**
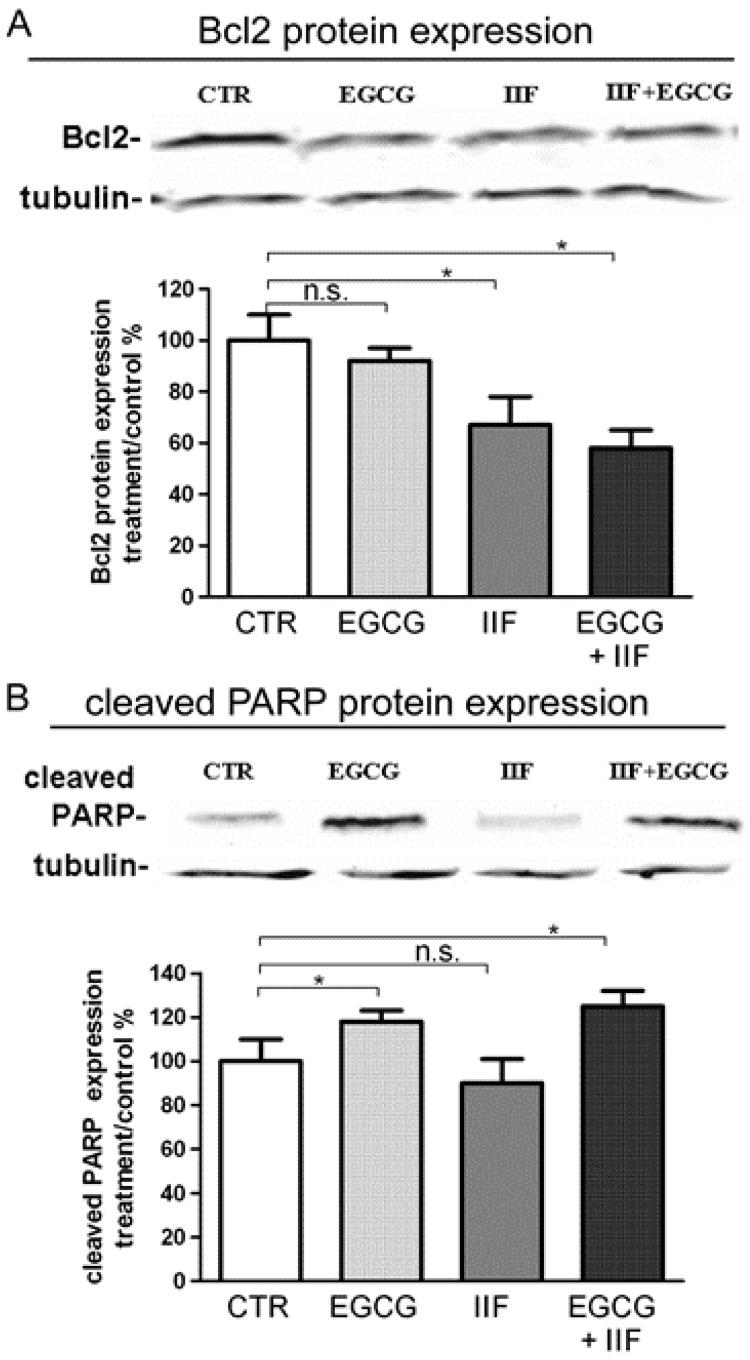
Effect of EGCG and IIF treatments on apoptosis-related proteins in BE(2)-C neuroblastoma cells. Modulation of Bcl-2 and cleaved PARP levels after 24 h individual and combined 20 μg/mL EGCG and 10 μM IIF treatments. Proteins (50 μg) from total cell lysates were subjected to SDS–PAGE and Western blot analysis while using Bcl-2 (**A**) and cleaved PARP antibodies (**B**). The values were normalized to the untreated controls. β-tubulin was used as a loading control. The results are expressed as the average ± SE of three independent experiments. * *p* < 0.05. n.s.: not significant.

After 72 h exposure to individual IIF and EGCG doses, the cell viability decreased to 62% (20 μg/mL EGCG) and 52% (20 μM IIF), respectively (Figure 3A,B). Combination treatments resulted in greater cytotoxicity, even using lower IIF concentrations: 10 μM IIF and 20 μg/mL EGCG given in combination to BE(2)-C cells for 72 h lowered cell viability to 26% (Figure 3C). Synergism was found using 10 μM IIF and 20 μg/mL EGCG: these concentrations were used for all of the subsequent experiments (Table S3). Increased cytotoxicity and inhibition of cell proliferation were associated with apoptosis, as demonstrated by Bax, Bcl-2, and PARP Western blot analysis. In our hands, Bax was nearly undetectable (data not shown), possibly related to apoptosis pathway dysregulation [20] or p53 missense mutation at codon 135 found in the BE(2)-C cell line [21,22]. A Bcl-2 decrease was clearly detected and it was primarily achieved by IIF activity, whereas PARP cleavage was mainly due to the EGCG effect (Figure 4), a finding that does not reflect synergism but diverse activity on the apoptosis pathway.

### 3.3. EGCG and IIF Downregulated N-MYC Expression 

**Figure 5:**
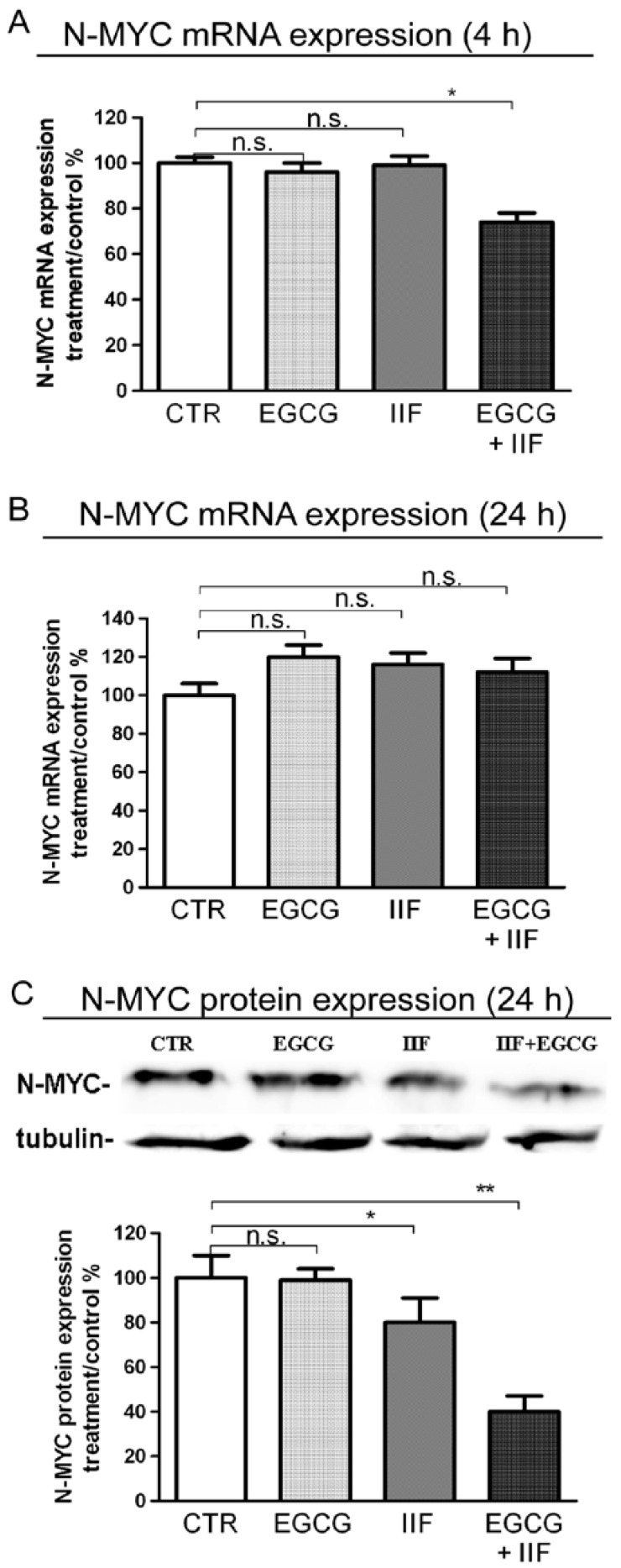
Downregulation of N-MYC expression by EGCG and IIF treatments in BE(2)-C neuroblastoma cells. Cells were treated with 20 μg/mL EGCG and 10 μM IIF, individually and in combination. N-MYC qPCR analysis in control and treated cells after 4 h (**A**) and 24 h (**B**). GAPDH was used as a control. (**C**) Proteins (50 μg) from total cell lysates were subjected to SDS–PAGE and Western blot analysis of N-MYC expression after 24 h treatments. The values were normalized to the untreated controls. β-tubulin was used as a loading control. The results are expressed as the average ± SE of three independent experiments. * *p* < 0.05; ** *p* < 0.01. n.s.: not significant. GAPDH: Glyceraldehyde 3-phosphate dehydrogenase.

N-MYC amplification and overexpression is the best-characterized genetic marker of risk in neuroblastoma. N-MYC is expressed in neural cells during development and is downregulated along with differentiation. In neuroblastoma, N-MYC amplification and/or overexpression play multiple roles in malignancy and maintenance of a stem-like state, as they can activate the transcription of genes that are involved in metastasis, survival, proliferation, pluripotency, self-renewal, and angiogenesis. We treated BE(2)-C cells with EGCG and IIF, evaluating N-MYC expression by q-PCR and Western blot. A significant mRNA N-MYC decrease was only found after 4 h EGCG+IIF treatment (Figure 5A,B). In contrast, N-MYC protein expression was dramatically reduced after combined treatment for 24 h (Figure 5C), a finding that might be due to synergism. Therefore, EGCG+IIF treatment was effective in reducing the N-MYC protein level in BE(2)-C cells.

### 3.4. Molecular Targets of EGCG and IIF and N-MYC Downregulation

**Figure 6:**
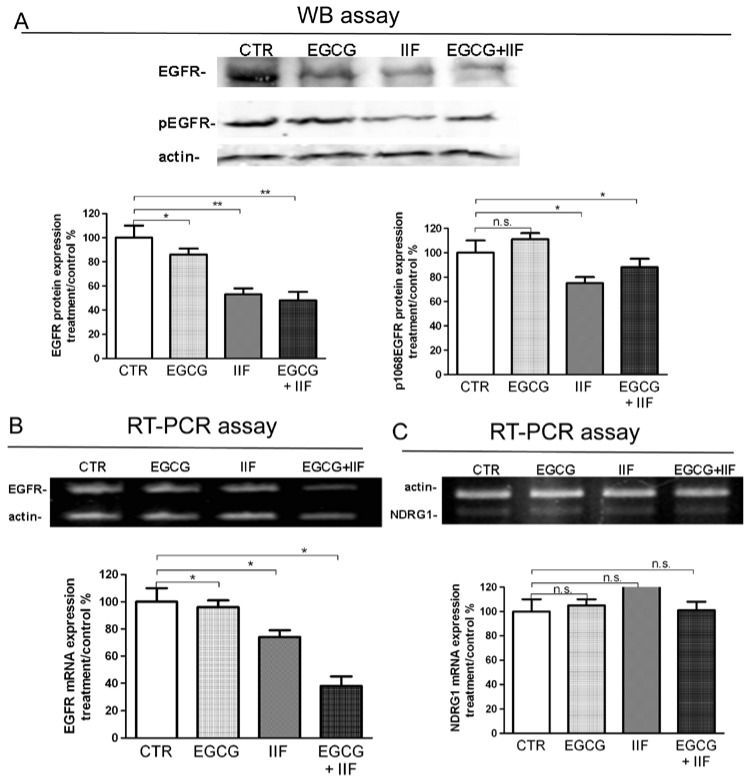
Downregulation of EGFR and p1068EGFR expression by EGCG and IIF treatments in BE(2)-C neuroblastoma cells. Cells were treated with 20 μg/mL EGCG and 10 μM IIF, individually and in combination for 24 h. (**A**) Proteins (50 μg) from total cell lysate were subjected to SDS–PAGE and Western blot analysis of EGFR and p1068EGFR expression after 24 h treatments. Actin was used as a loading control. RT-PCR analysis of EGFR (**B**) and NDRG1 (**C**) in control and treated cells. β-actin was used as a control. The values were normalized to the untreated controls. The results are expressed as the average ± SE of three independent experiments. * *p* < 0.05; ** *p* < 0.01. n.s.: not significant. WB: western blot; RT-PCR: reverse transcriptase-polymerase chain reaction.

EGFR is a gene that is directly downregulated by N-MYC [23], which is often overexpressed in neuroblastoma, and previously demonstrated to be a molecular target of both EGCG and IIF [12,14]. EGFR expression was detected in control BE(2)-C cells: protein expression was reduced as was p1068 phosphorylation (Figure 6A) in keeping with a significant decrease of RNA expression after both individual and combined treatments (Figure 6B). N-MYC and EGFR expression are reciprocally related with N-MYC downregulated gene 1 (NDRG1). NDRG1 is a transcription factor that is implicated in growth arrest, cell differentiation, and response to hypoxia, and it is regarded as an anti-metastatic molecule in prostate carcinoma [24]. Furthermore, NDRG1 can be upregulated by retinoic acid. N-MYC downregulation was found to induce re-expression of NDRG1 that, in turn, can repress the HER family oncogenes, including EGFR [25]. We found that NDRG1 was expressed in untreated BE(2)-C cells: after 24 h, treatments no significant change was detected (Figure 6C).

### 3.5. EGCG and IIF Limited Invasion and Metastatic Capability

N-MYC overexpression contributes to all phases, leading to metastasis: loss of cell adhesion, increased motility, invasion, and degradation of surrounding matrices. Our previous studies on human carcinoma demonstrated that the EGCG+IIF combination was very effective in decreasing the expression of MMP-2 and MMP-9, which are molecular markers of invasion. 

**Figure 7:**
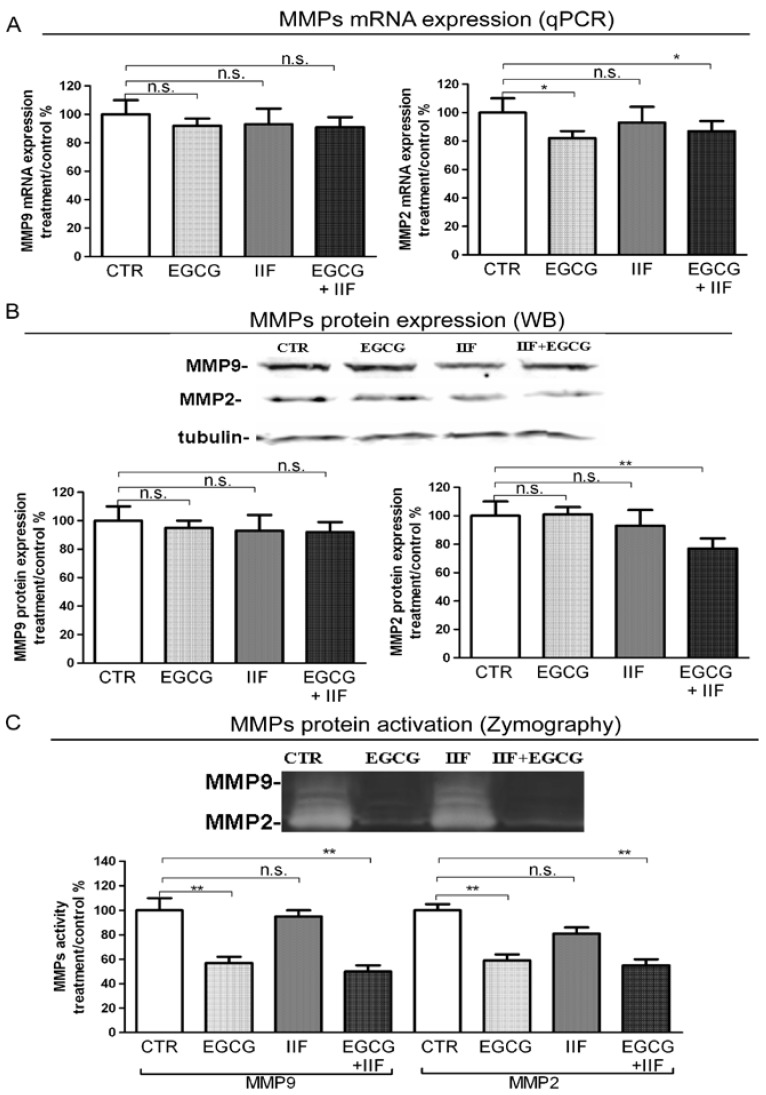
MMP-2 and MMP-9 expression and activity after EGCG and IIF treatments in BE(2)-C neuroblastoma cells. Cells were treated with 20 μg/mL EGCG and 10 μM IIF, individually and in combination for 24 h. (**A**) MMP-2 and MMP-9 qPCR was performed after RNA isolation and GAPDH was used as an internal control. (**B**) Western blot analysis of MMP-2 and MMP-9. The values were normalized to the untreated controls. β-tubulin was used as a loading control. (**C**) Zymography analysis of MMP-2 and MMP-9. Cells were seeded and after 18 h placed in serum-free medium with EGCG (20 μg/mL) or IIF (10 μM) or both for 24 h. MMP activity is indicated by clear bands. The results are expressed as the average ± SE of three independent experiments. * *p* < 0.05; ** *p* < 0.01. n.s.: not significant. MMP-2: Metalloproteinase-2 (MMP-2); MMP-9: Metalloproteinase-9.

**Figure 8:**
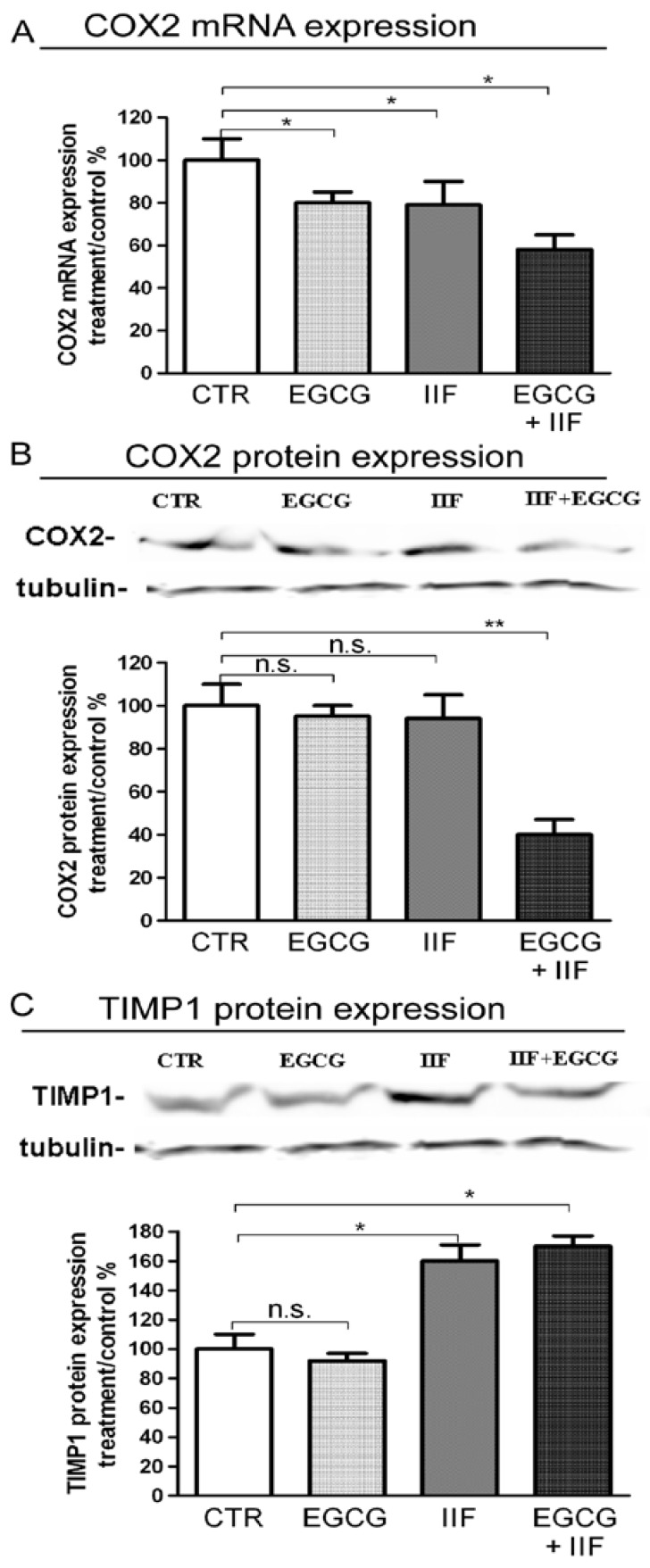
COX-2 and TIMP-1 expression after EGCG and IIF treatments in BE(2)-C neuroblastoma cells. (**A**) Cells were treated with 20 μg/mL EGCG and 10 μM IIF, individually and in combination for 24 h. (**A**) qPCR of COX-2 expression was performed after RNA isolation. GAPDH was used as an internal control. Western blot analysis of COX-2 (**B**) and TIMP-1 (**C**). The values were normalized to the untreated controls. β-tubulin was used as a loading control. The results are expressed as the average ± SE of three independent experiments. * *p* < 0.05; ** *p* < 0.01. n.s.: not significant.

MMP-2 and MMP-9 qPCR showed that MMP-2 mRNA and protein expression were significantly lowered by EGCG+IIF treatments (Figure 7A,B). To further investigate the functional activity of MMP-2 and 9, we turned to zymography, which detects MMP activity. We found that both EGCG and EGCG+IIF-treated samples showed around 50% MMP-2 and MMP-9 inhibition (Figure 7C): so EGCG resulted more active than IIF in inhibiting MMP activity. In parallel, TIMP-1, which negatively regulates MMP-9, was upregulated (Figure 8C). We investigated the effects on COX-2, a molecule that is associated with osteolytic bone metastasis in neuroblastoma [26], which promotes MMP expression. We observed that COX2 mRNA and protein expression were also downregulated (Figure 8A,B): in this case, a synergistic effect may be speculated. We concluded that, in addition to N-MYC downregulation, EGCG+IIF treatment attenuated the biopathological features of metastatic potential.

### 3.6. EGCG and IIF Impaired Sphere Formation in BE(2)-C Cells

**Figure 9:**
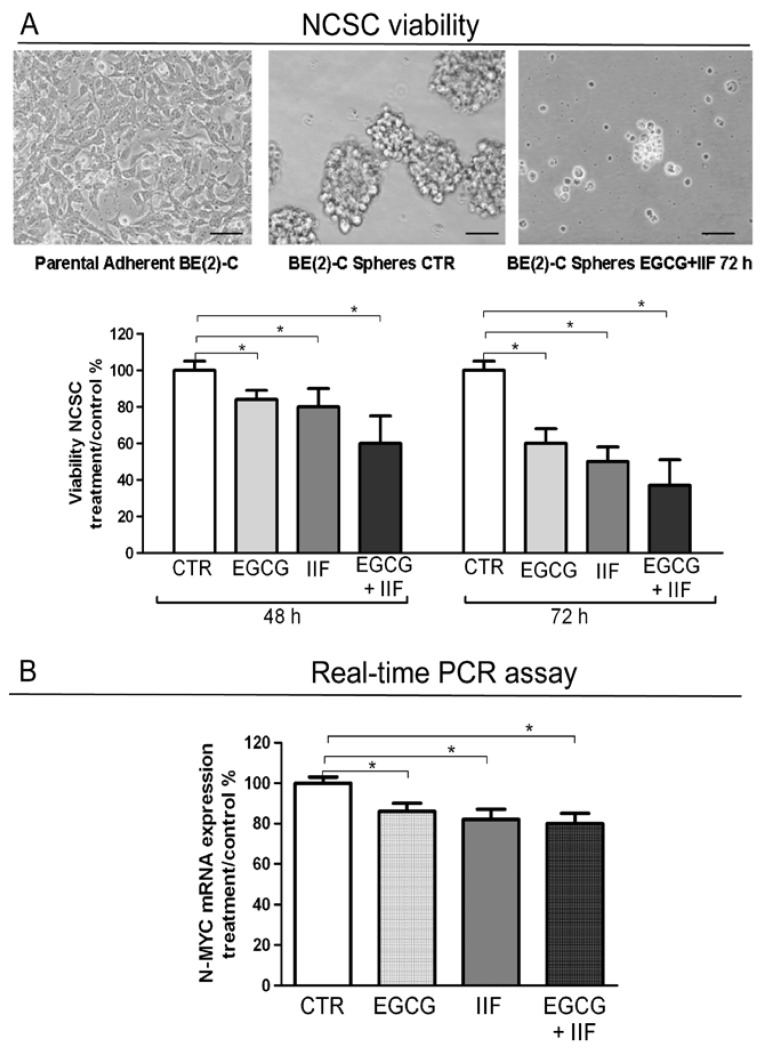
EGCG and IIF treatments limited sphere formation and N-MYC expression in BE(2)-C neuroblastoma cells. (**A**) Neural cancer stem cells (NCSC). Parental adherent BE(2)-cells were grown in non-adherent serum-free conditions to develop spheres for 72 h (CTR) and treated with 20 μg/mL EGCG and 10 μM IIF, individually and in combination for 72 h. Viability was evaluated by Trypan blue assay. (**B**) N-MYC mRNA expression was evaluated by qPCR in control (CTR) and treated cells. GAPDH was used as a loading control. The results are expressed as the average ± SE of three independent experiments. * *p* < 0.05. n.s.: not significant.

Neuroblastoma is a stem-like cell disease: neuroblastoma cancer cells can undergo tumor sphere transformation when grown under serum-free conditions. We investigated the effects of EGCG and IIF treatments on BE(2)-C sphere formation and viability, as evaluated by Trypan blue assay. In the control samples, spheres were clearly detected after 48 h. Incubation with EGCG and IIF at 48 h and 72 h reduced the sphere number and size (Figure 9A). Trypan blue assay demonstrated a cytotoxic effect: after 72 h treatment viability decreased to 50%. N-MYC gene expression significantly decreased after all the treatments (Figure 9B). We therefore concluded that individual and combined treatments with EGCG and IIF impaired sphere formation and increased cytotoxicity: a synergistic effect was detected after 48 h of treatment (Table S4).

## 4. Discussion 

We studied the effects of EGCG and IIF on BE(2)-C, a neuroblastoma cell line representative of high-risk neuroblastoma patients, and on BE(2)-C-spheres, which is a derivative BE(2)-C subpopulation, grown in low attachment conditions and serum-free medium, thought to correspond to cancer stem cells. We found a significant arrest of cell growth together with downregulation of N-MYC expression and molecular markers of invasion. Furthermore, the EGCG and IIF combination was cytotoxic: sphere formation was impaired and cell death occurred in a significant percentage of cells after 48 and 72 h. Overall, these findings demonstrated the efficacy of EGCG and IIF to limit neuroblastoma cell growth.

EGCG and green tea catechins are considered powerful antioxidant and chemopreventive molecules. They have been found to serve as antioxidants and improve the detoxification system, thereby inhibiting carcinogen metabolism and cancerogenesis. Furthermore, they limit cancer cell proliferation and tumor-initiating cell self-renewal by modulating the numerous molecules fundamental for cancer onset and progression [27]. Retinoids are used in neuroblastoma therapy: high-risk neuroblastoma patients are treated with 13cRA in postconsolidation therapy after autologous hematopoietic stem cell transplantation [5]. Retinoids are capable of inducing differentiation at low doses, but many high-risk neuroblastoma patients do not respond to 13cRA treatment [4]. Adverse effects were not severe, but better results were achieved while combining 13cRA with immunotherapy [28] or the histone deacetylase inhibitor Vorinostat [6]. The combination of ATRA, 13-cRA, and 4-HPR with EGCG was investigated on SH-SY5Y cells, which is a neuroblastoma cell line lacking N-MYC amplification and overexpression, resulting in cell growth inhibition, apoptosis and N-MYC protein decrease [16]. IIF is a synthetic and safe derivative of Vitamin A, used as a food supplement, which demonstrated enhanced anti-neoplastic effects in neuroblastoma and various cancer cell lines [29]. When IIF was given in combination with EGCG to breast and colorectal carcinoma cell lines, cytotoxicity increased with downregulation of the molecular markers of neoplastic progression [12,13,14]. In BE(2)-C cells, EGCG+IIF treatment downregulated N-MYC protein expression, whereas mRNA was reduced only after 4h EGCG+IIF treatment. N-MYC is considered to be the most prominent molecular marker in neuroblastoma therapy, but it is not a directly targetable molecule [30]. N-MYC expression is high during early developmental stages then gradually subsides as the neural crest precursors differentiate into sympathetic neurons. Aberrantly high N-MYC is thought to contribute to neuroblastoma development, at least in part, by promoting a persistent mesenchymal phenotype within neuroblastoma cells. Strategies to circumvent N-MYC activity include the targeting regulators of N-MYC mRNA and protein stability, and differentiation agents [10]. We found that EGCG and IIF decreased N-MYC protein expression, with a modest impact on mRNA expression after 4h treatment, in keeping with data obtained using other molecules regulating N-MYC protein stability by different mechanisms (LY294002, BEZ235, AURAKA ligands) [10]. Indeed, a significant inhibition of N-MYC mRNA expression was found after 72 h EGCG and IIF treatments of the BE(2)-C sphere. This discrepancy might be attributed to different treatment time and cell population analyzed. NSCS is a minority subset of the tumor cell population, which is thought to be rich in tumor-initiating cells [31] considered responsible for drug resistance, local relapse and metastatic spread. N-MYC promotes a stem-like state by impairing the differentiation pathways and supporting self-renewal and pluripotency. N-MYC-amplified neuroblastoma cell lines, including BE(2)-C cells, form spheres more frequently than non-N-MYC-amplified cell lines and sphere formation is sensitive to cellular differentiation status [32]. In the present study, BE(2)-C sphere formation was reduced and associated with cell death after EGCG and IIF treatments. In addition, the EGCG and IIF combination was effective in limiting cell growth in both adherent BE(2)-C cells and the BE(2)-C sphere, possibly by downregulation of N-MYC. We cannot exclude that EGCG and IIF treatments might act in adherent and sphere BE(2)-C cells by different mechanisms: N-MYC acts in the context of a large protein network. A specific subset of genes might regulate N-MYC in the stem-like or lineage committed pattern. This point needs to be clarified and it goes beyond the aim of the present study, but this finding makes EGCG and IIF molecules potentially useful in patients in the remission phase, as they might limit and kill the tumor-initiating cells that are responsible for relapse and therapeutic failure.

The efficacy of EGCG and IIF treatments against BE-(2)-C cells extended beyond their cytotoxic activity to include changes and downregulation of molecules that are associated with the invasive phenotype. In some cases, this effect was clearly synergistic (MTT, N-MYC, and COX2 protein downregulation); in others, the individual effects of EGCG or IIF predominated. Whereas, IIF belongs to class of molecules that are known to target a specific signaling pathway, EGCG is a polytarget molecule that is able to down or upregulate numerous different molecules. We can speculate that, in some cases, the final effects resulted from an independent regulation of different molecules. In human neuroblastoma cells, N-MYC and Bcl-2 co-expression induced MMP-2 secretion and activation [33]. In the present study, zymography demonstrated significant inactivation of both MMP-2 and MMP-9. The EGCG+IIF treatment was not more effective than individual EGCG treatments, as IIF did not significantly reduce MMP-2 and -9 activity. Likewise, the expression of TIMP-1, which is a negative regulator of MMP-9, increased after IIF and EGCG+IIF treatments, a finding that explains the downregulation of MMP-9 activity. The combined EGCG+IIF treatment also downregulated COX-2, which is a strong pro-inflammatory molecule that enhances MMP expression and activity.

The major signaling pathways leading to uncontrolled tumor growth and aggressive behavior in high-risk neuroblastoma patients are related to a small number of altered genes, N-MYC being one of the most important. With respect to many solid tumors, primary neuroblastoma shows an unexpectedly low genetic complexity [34], but the outcome in high-risk patients remains dismal. EGFR is often expressed in neuroblastoma, but there is no consensus on its role and prognostic impact [35]. In mice, EGF (and EGFR) is a key molecule in sympathoadrenal progenitor migration to either the analogue of the paravertebral sympathetic ganglia or adrenal medulla/suprarenal sympathetic ganglia. Different cell origin might explain the distinct worse clinical presentations of adrenal neuroblastoma when compared to non-adrenal derivative neoplasm [36]. A few neuroblastoma patients proved responsive to EGFR inhibitors such as Gefitinib, but only in association with other drugs [37]. In breast carcinoma, we demonstrated that EGFR is a molecular target of both EGCG and IIF, but the present study failed to find a relation between N-MYC and EGFR in BE(2)-C cells. Although IIF induced a significant increase in mRNA NDRG1 expression and EGFR (mRNA, total protein, and p1068EGFR) decreased, the real meaning and role of EGFR activity in neuroblastoma require further investigation. 

In conclusion, an RXR agonist (IIF) that is associated with a polytarget molecule (EGCG) might be a promising adjunct to improve the outcome of retinoid treatment. Both of the molecules are used as food supplements, lack side-effects, and are considered beneficial for human health. EGCG, in particular, has been widely investigated in healthy volunteers ruling out toxic effects. Our previous study also found no cytotoxic effect on normal human peripheral blood lymphocytes after EGCG treatments [12]. IIF was not toxic in animals [38] and it is now sold as a food supplement in the USA. High-risk neuroblastoma patients who survive and do not relapse may experience long-term toxic effects, chronic diseases and increased risk of tumor recurrence. In the search for less aggressive but efficient therapies, the EGCG and IIF combination is a promising candidate.

## Supplementary Materials

The following are available online at http://www.mdpi.com/2072-6643/10/9/1141/s1, Table S1. Primer sequences for RT-PCR. Table S2. Primer sequences for qPCR. Figure S1: PPAR and PPAR mRNA expression in BE(2)-C neuroblastoma cells. Table S3. Combination Index (CI). Table S4. Combination index (CI).
