# Supplementary material for: Epigallocatechin-3-gallate and 6-OH-11-O-Hydroxyphenanthrene Limit BE(2)-C Neuroblastoma Cell Growth and Neurosphere Formation In Vitro
Source: Nutrients. 2018 Aug 22;10(9):1141. doi: 10.3390/nu10091141 (PMC6164794; doi:10.3390/nu10091141)
Supplement: Supplementary file 1 [file nutrients-10-01141-s001.zip › Supplementary Table 1.pdf]

Supplementary **Table 1.** Primer sequences for RT-PCR

**Gene**	**Sequence**	**Annealing t °C**	**Base pair**
**β-actin**	F5'-ATCGTGCGTGACATTAAGGAGAAG-3'	60	179
	R5'-AGGAAGGAAGGCTGGAAGAGTG-3'		
**EGFR**	F5'- CTGACGCAGTTGGGCACTTT-3'	60	301
	R5'-TCATGGGCAGCTCCTTCAGT-3'		
**NDRG1**	F5'-CTCTGTTCACGTCACGCTGT-3'	60	104
	R5′-AGAGGGGGTTGTAGCAGGTT-3′		
