# Supplementary material for: Epigallocatechin-3-gallate and 6-OH-11-O-Hydroxyphenanthrene Limit BE(2)-C Neuroblastoma Cell Growth and Neurosphere Formation In Vitro
Source: Nutrients. 2018 Aug 22;10(9):1141. doi: 10.3390/nu10091141 (PMC6164794; doi:10.3390/nu10091141)
Supplement: Supplementary file 1 [file nutrients-10-01141-s001.zip › Supplementary Table 2.pdf]

Supplementary **Table 2.** Primer sequences for qPCR

**Gene**	**Sequence**	**Annealing t °C**	**Base pair**
**RXRα**	F5′-CAAGGACTGCCTGATTGACA-3	56	145
	R5′-CGACTCCACCTCATTCTCGT-3′		
**RXRβγ**	F5'-GGTTTGCCAAGCTGCTGCT-3	56	122
	R5′-CATCTCCATGAGGAAGGTGT-3′		
**GAPDH**	F5′-GCAGGGATGATGTTCTGGAG-3′	56	123
	R5′-TGGTATCGTGGAAGGACTCATGAC-3		
**RARα**	F5'-TGGGTGGACTCTCCCCGCCA-3'	60	460
	R5'-CCCACCTCCGGCGTCAGCGTG-3'		
**RARβ**	F5'- CACTGGCTTGACCATCGCAGACC-3'	64	481
	R5'- GAGAGGTGGCATTGATCCAGG-3'		
**RARγ**	F5'- GGCCTGGGCCAGCCTGACCTC-3'	64	537
	R5'- GAGCCCCAGATCCAGCTGCACG-3'		
**N-MYC**	F5'- GTCACCACATTCACCATCAC-3'	60	96
	R5'- GGGAAGGCATCGTTTGAG-3'		
**MMP-2**	F5'-ATCACATACAGGATCATTGGCTAC-3'	63	139
	R5'- TGATGTCTGCCTCTCCATCA-3'		
**MMP-9**	F5'-ACGCCGCTCACCTTCACTC-3'	63	180
	R5'-GGACCACAACTCGTCATCGTC-3'		
**COX-2**	F5'-CCTGTGCCTGATGATTGC-3'	60	162
	R5'-CTGATGCGTGAAGTGCTG		
**Actin**	F5'-GGCATCCACGAAACTACCTTCAAC-3'	60	138
	R5'-AGTGATCTCCTTCTGCATCCTGTC-3'		
