# Supplementary material for: Epigallocatechin-3-gallate and 6-OH-11-O-Hydroxyphenanthrene Limit BE(2)-C Neuroblastoma Cell Growth and Neurosphere Formation In Vitro
Source: Nutrients. 2018 Aug 22;10(9):1141. doi: 10.3390/nu10091141 (PMC6164794; doi:10.3390/nu10091141)
Supplement: Supplementary file 1 [file nutrients-10-01141-s001.zip › Supplementary Table 3.pdf]

Supplementary **Table 3.** Combination Index (CI)

	IIF 10	**Time**
EGCG 20	>1	24 h
EGCG 20	**<1**	48 h
EGCG 20	**<1**	72 h

Supplementary Table 3. Synergy evaluation of EGCG (µg/mL) and IIF (µM) treatment effects with the combination index (CI). MTT assay data in BE(2)-C cells, after 24-48-72 h treatment, were used to calculate additive (=1), synergistic (<1) or antagonistic (>1) effects as described in Materials and Methods**.**
