# Supplementary material for: Epigallocatechin-3-gallate and 6-OH-11-O-Hydroxyphenanthrene Limit BE(2)-C Neuroblastoma Cell Growth and Neurosphere Formation In Vitro
Source: Nutrients. 2018 Aug 22;10(9):1141. doi: 10.3390/nu10091141 (PMC6164794; doi:10.3390/nu10091141)
Supplement: Supplementary file 1 [file nutrients-10-01141-s001.zip › Supplementary Table 4.pdf]

Supplementary **Table 4.** Combination index (CI)

	IIF 10	**Time**
EGCG 20	**<1**	48 h
EGCG 20	>1	72 h

Supplementary Table 4: Synergy evaluation of EGCG (μg/mL) and IIF (μM) treatment effects with the combination index (CI). Vitality was assayed by Trypan blue in NCSC after 48 and 72 h treatment. Additive (=1), synergistic (<1) or antagonistic (>1) effects were evaluated as described in Materials and Methods.
